# Supplementary figures and images for: Imprinted Grb10, encoding growth factor receptor bound protein 10, regulates fetal growth independently of the insulin-like growth factor type 1 receptor (Igf1r) and insulin receptor (Insr) genes
Source: BMC Biol. 2024 May 30;22:127. doi: 10.1186/s12915-024-01926-w (PMC11140863; doi:10.1186/s12915-024-01926-w)

Fig. S1

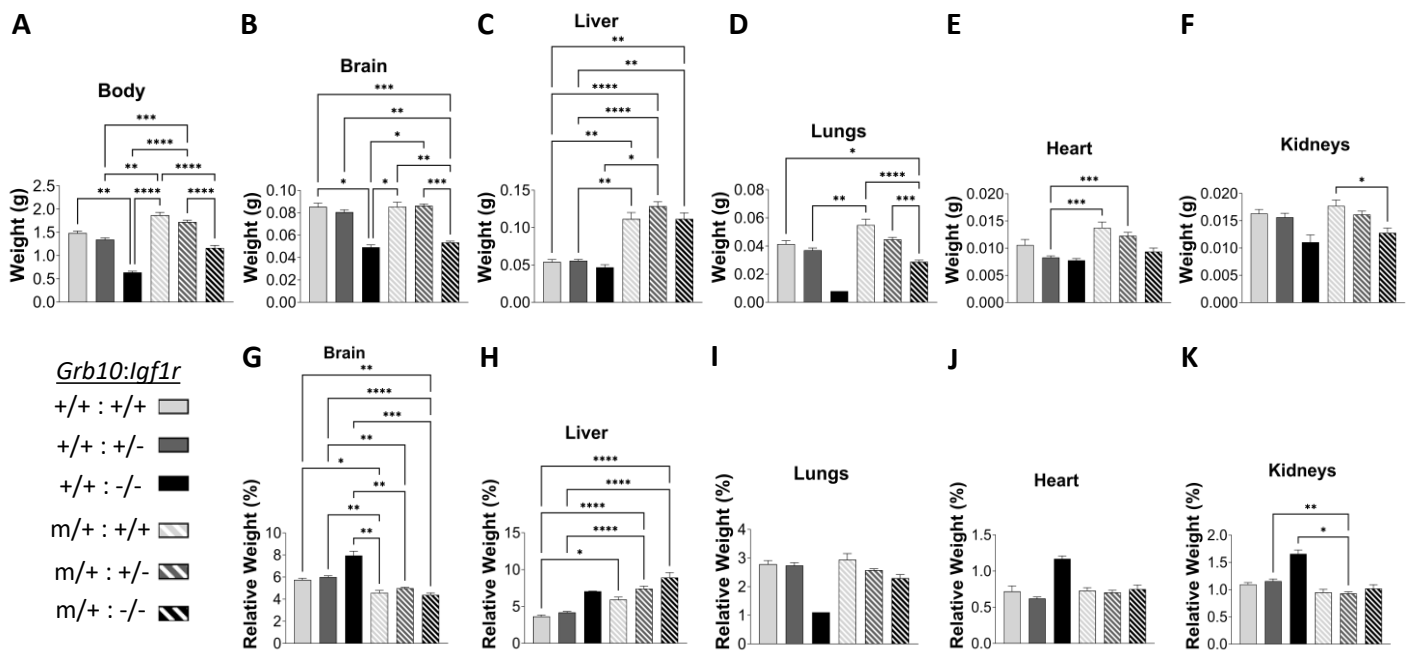

Fig. S2

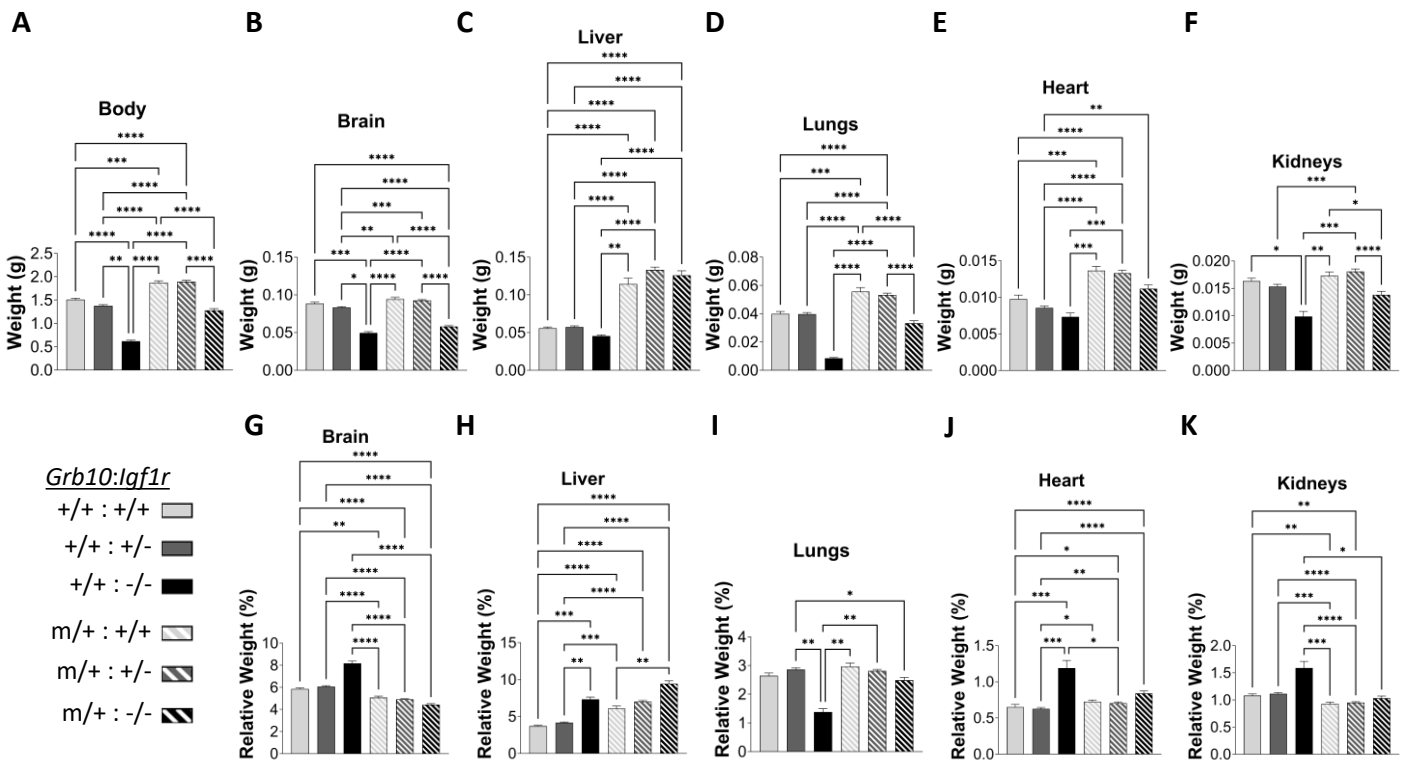

Fig. S3

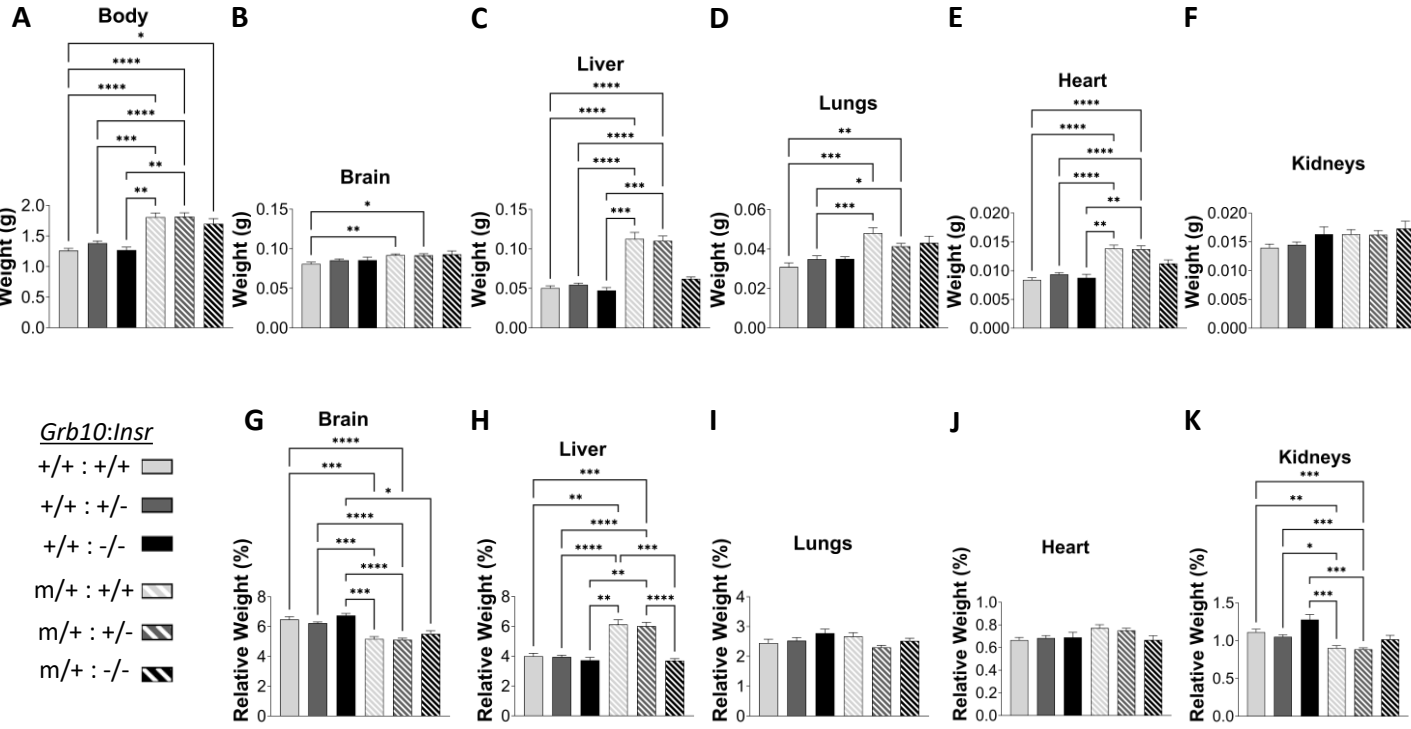

Fig. S4

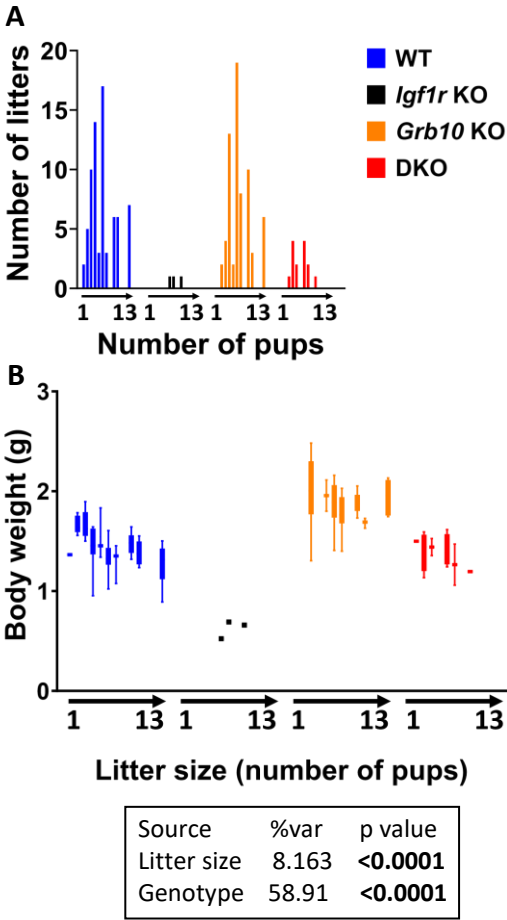

Supplement: Supplementary file 1 — Additional file 1: Figures S1-S4. Fig. S1. Weights at PN1 from progeny of crosses between Grb10ins7 KO and Igf1r KO mice. Body weights are shown for the six offspring genotypes (A). Actual weights of brain (B), liver (C), lungs (D), heart (E) and kidneys (F) are shown alongside relative weights of the same organs, expressed as a percentage of body mass (G-K). Values represent means and SEM, tested using ANOVA with Kruskal-Wallis post hoc statistical tests. Sample sizes were, for body, Grb10 wild type (WT):Igf1r WT n=15, Grb10 WT:Igf1r Het n=23, Grb10 KO:Igf1r WT n=8, Grb10 KO:Igf1r Het n=18, Grb10 WT:Igf1r KO n=7, Grb10:Igf1r DKO n=12; brain, Grb10 WT:Igf1r WT n=15, Grb10 WT:Igf1r Het n=23, Grb10 KO:Igf1r WT n=8, Grb10 KO:Igf1r Het n=17, Grb10 WT:Igf1r KO n=3, Grb10:Igf1r DKO n=8 liver, Grb10 WT:Igf1r WT n=15, Grb10 WT:Igf1r Het n=23, Grb10 KO:Igf1r WT n=8, Grb10 KO:Igf1r Het n=17, Grb10 WT:Igf1r KO n=2, Grb10:Igf1r DKO n=7; lungs, Grb10 WT:Igf1r WT n=15, Grb10 WT:Igf1r Het n=23, Grb10 KO:Igf1r WT n=8, Grb10 KO:Igf1r Het n=17, Grb10 WT:Igf1r KO n=1, Grb10:Igf1r DKO n=7; heart, Grb10 WT:Igf1r WT n=14, Grb10 WT:Igf1r Het n=23, Grb10 KO:Igf1r WT n=8, Grb10 KO:Igf1r Het n=17, Grb10 WT:Igf1r KO n=2, Grb10:Igf1r DKO n=7; kidneys, Grb10 WT:Igf1r WT n=15, Grb10 WT:Igf1r Het n=23, Grb10 KO:Igf1r WT n=8, Grb10 KO:Igf1r Het n=17, Grb10 WT:Igf1r KO n=2, Grb10:Igf1r DKO n=7. Asterisks indicate p-values, * p <0.05, ** p <0.01, *** p <0.001, **** p<0.0001. Fig. S2. Weights at PN1 from progeny of crosses between Grb10Δ2-4 KO and Igf1r KO mice. Body weights are shown for the six offspring genotypes (A). Actual weights of brain (B), liver (C), lungs (D), heart (E) and kidneys (F) are shown alongside relative weights of the same organs, expressed as a percentage of body mass (G-K). Values represent means and SEM, tested using ANOVA with Kruskal-Wallis post hoc statistical tests. Sample sizes were, for body, Grb10 WT:Igf1r WT n=35, Grb10 WT:Igf1r Het n=69, Grb10 KO:Igf1r WT n=25, Grb10 [file 12915_2024_1926_MOESM1_ESM.pdf]
