# Supplementary material for: Imprinted Grb10, encoding growth factor receptor bound protein 10, regulates fetal growth independently of the insulin-like growth factor type 1 receptor (Igf1r) and insulin receptor (Insr) genes
Source: BMC Biol. 2024 May 30;22:127. doi: 10.1186/s12915-024-01926-w (PMC11140863; doi:10.1186/s12915-024-01926-w)
Supplement: Supplementary file 2 — Additional file 2: Tables S1-S3. Table S1. Chi-squared statistical tests of offspring survival from crosses involving Grb10 KO and Igf1r KO strains. Offspring collected from crosses between Grb10Δ2-4+/p: Igfr1+/- females and Grb10Δ2-4+/+: Igfr1+/- males at, (A) PN1 and (B) e17.5. (C) Offspring collected at PN1 from crosses between Grb10ins7+/p: Igf1+/- females and Grb10ins7+/+: Igf1+/- males. Deviation from the expected Mendelian ratio was considered significant at p<0.05. PN1: Grb10Δ2-4+/p: Igf1r+/- x Grb10Δ2-4+/+: Igf1r+/- e17.5: Grb10Δ2-4+/p: Igf1r+/- x Grb10Δ2-4+/+: Igf1r+/-. Table S2. Chi-squared statistical tests of offspring survival from crosses between the Grb10 Δ2-4 KO and Insr KO strains. Offspring collected from crosses between Grb10Δ2-4+/p: Insr+/- females and Grb10Δ2-4+/p: Insr+/- males at PN1 (A) and at e17.5 (B). Deviation from the expected Mendelian ratio was considered significant at p<0.05. Table S3. Litter size information for progeny of mouse crosses involving Grb10Δ2-4 and either Igf1r KO or Insr KO. The number of pups per litter is shown as a range and mean for each dataset. [file 12915_2024_1926_MOESM2_ESM.docx]

**Table S1.**

**A)** Chi-square test **(**PN1): *Grb10Δ2-4^+/p^*: *Igf1r^+/-^* x *Grb10Δ2-4^+/+^*: *Igf1r^+/-^*

| Chi-square | 13.73 |  |  |  |
| --- | --- | --- | --- | --- |
| DF | 5 |  |  |  |
| P value (two-tailed) | 0.0174 |  |  |  |
| P value summary | * |  |  |  |
| Is discrepancy significant (P < 0.05)? | Yes |  |  |  |
|  |  |  |  |  |
| Outcome | Expected # | Observed # | Expected % | Observed % |
| *Grb10^+/+^:Igf1r^+/+^* | 29.63 | 35 | 12.5 | 14.77 |
| *Grb10^m/+^:Igf1r^+/+^* | 29.63 | 25 | 12.5 | 10.55 |
| *Grb10^+/+^:Igf1r^+/-^* | 59.25 | 69 | 25 | 29.11 |
| *Grb10^m/+^:Igf1r^+/-^* | 59.25 | 67 | 25 | 28.27 |
| *Grb10^+/+^:Igf1r^-/-^* | 29.63 | 13 | 12.5 | 5.485 |
| *Grb10^m/+^:Igf1r^-/-^* | 29.63 | 28 | 12.5 | 11.81 |
| TOTAL | 237 | 237 | 100 | 100 |

**B)** Chi-square test (e17.5): *Grb10Δ2-4^+/p^*: *Igf1r^+/-^* x *Grb10Δ2-4^+/+^*: *Igf1r^+/-^*

| Chi-square | 14.11 |  |  |  |
| --- | --- | --- | --- | --- |
| DF | 5 |  |  |  |
| P value (two-tailed) | 0.015 |  |  |  |
| P value summary | * |  |  |  |
| Is discrepancy significant (P < 0.05)? | Yes |  |  |  |
|  |  |  |  |  |
| Outcome | Expected # | Observed # | Expected % | Observed % |
| *Grb10^+/+^:Igf1r^+/+^* | 4.75 | 10 | 12.5 | 26.32 |
| *Grb10^m/+^:Igf1r^+/+^* | 4.75 | 3 | 12.5 | 7.895 |
| *Grb10^+/+^:Igf1r^+/-^* | 9.5 | 7 | 25 | 18.42 |
| *Grb10^m/+^:Igf1r^+/-^* | 9.5 | 8 | 25 | 21.05 |
| *Grb10^+/+^:Igf1r^-/-^* | 4.75 | 1 | 12.5 | 2.632 |
| *Grb10^m/+^:Igf1r^-/-^* | 4.75 | 9 | 12.5 | 23.68 |
| TOTAL | 38 | 38 | 100 | 100 |

**C)** Chi-square test (PN1): *Grb10ins7^+/p^*: *Igf1r^+/-^* x *Grb10ins7^+/+^*: *Igf1r^+/-^*

| Chi-square | 4.566 |  |  |  |
| --- | --- | --- | --- | --- |
| DF | 5 |  |  |  |
| P value (two-tailed) | 0.4711 |  |  |  |
| P value summary | ns |  |  |  |
| Is discrepancy significant (P < 0.05)? | No |  |  |  |
|  |  |  |  |  |
| Outcome | Expected # | Observed # | Expected % | Observed % |
| *Grb10^+/+^:Igf1r^+/+^* | 10.38 | 15 | 12.5 | 18.07 |
| *Grb10^m/+^:Igf1r^+/+^* | 10.38 | 8 | 12.5 | 9.639 |
| *Grb10^+/+^:Igf1r^+/-^* | 20.75 | 23 | 25 | 27.71 |
| *Grb10^m/+^:Igf1r^+/-^* | 20.75 | 18 | 25 | 21.69 |
| *Grb10^+/+^:Igf1r^-/-^* | 10.38 | 7 | 12.5 | 8.434 |
| *Grb10^m/+^:Igf1r^-/-^* | 10.38 | 12 | 12.5 | 14.46 |
| TOTAL | 83 | 83 | 100 | 100 |

**Table S2.**

1. Chi-square test (PN1): *Grb10Δ2-4^+/p^*: *Insr^+/-^* x *Grb10Δ2-4^+/p^*: *Insr^+/-^*

| Chi-square | 10.68 |  |  |  |
| --- | --- | --- | --- | --- |
| DF | 11 |  |  |  |
| P value (two-tailed) | 0.4702 |  |  |  |
| P value summary | ns |  |  |  |
| Is discrepancy significant (P < 0.05)? | No |  |  |  |
|  |  |  |  |  |
| Outcome | Expected # | Observed # | Expected % | Observed % |
| *Grb10^+/+^:Insr^+/+^* | 6.313 | 5 | 6.25 | 4.95 |
| *Grb10^+/p^:Insr^+/+^* | 6.313 | 7 | 6.25 | 6.931 |
| *Grb10^m/+^:Insr^+/+^* | 6.313 | 8 | 6.25 | 7.921 |
| *Grb10^m/p^:Insr^+/+^* | 6.313 | 10 | 6.25 | 9.901 |
| *Grb10^+/+^:Insr^+/-^* | 12.63 | 17 | 12.5 | 16.83 |
| *Grb10^+/p^:Insr^+/-^* | 12.63 | 13 | 12.5 | 12.87 |
| *Grb10^m/+^:Insr^+/-^* | 12.63 | 15 | 12.5 | 14.85 |
| *Grb10^m/p^:Insr^+/-^* | 12.63 | 11 | 12.5 | 10.89 |
| *Grb10^+/+^:Insr^-/-^* | 6.313 | 4 | 6.25 | 3.96 |
| *Grb10^+/p^:Insr^-/-^* | 6.313 | 2 | 6.25 | 1.98 |
| *Grb10^m/+^:Insr^-/-^* | 6.313 | 6 | 6.25 | 5.941 |
| *Grb10^m/p^:Insr^-/-^* | 6.313 | 3 | 6.25 | 2.97 |
| TOTAL | 101 | 101 | 100 | 100 |

**B)** Chi-square test (e17.5): *Grb10Δ2-4^+/p^*: *Insr^+/-^* x *Grb10Δ2-4^+/p^*: *Insr^+/-^*

| Chi-square | 18 |  |  |  |
| --- | --- | --- | --- | --- |
| DF | 11 |  |  |  |
| P value (two-tailed) | 0.0816 |  |  |  |
| P value summary | ns |  |  |  |
| Is discrepancy significant (P < 0.05)? | No |  |  |  |
|  |  |  |  |  |
| Outcome | Expected # | Observed # | Expected % | Observed % |
| *Grb10^+/+^:Insr^+/+^* | 7.75 | 10 | 6.25 | 8.065 |
| *Grb10^+/p^:Insr^+/+^* | 7.75 | 12 | 6.25 | 9.677 |
| *Grb10^m/+^:Insr^+/+^* | 7.75 | 11 | 6.25 | 8.871 |
| *Grb10^m/p^:Insr^+/+^* | 7.75 | 15 | 6.25 | 12.1 |
| *Grb10^+/+^:Insr^+/-^* | 15.5 | 16 | 12.5 | 12.9 |
| *Grb10^+/p^:Insr^+/-^* | 15.5 | 13 | 12.5 | 10.48 |
| *Grb10^m/+^:Insr^+/-^* | 15.5 | 13 | 12.5 | 10.48 |
| *Grb10^m/p^:Insr^+/-^* | 15.5 | 13 | 12.5 | 10.48 |
| *Grb10^+/+^:Insr^-/-^* | 7.75 | 4 | 6.25 | 3.226 |
| *Grb10^+/p^:Insr^-/-^* | 7.75 | 9 | 6.25 | 7.258 |
| *Grb10^m/+^:Insr^-/-^* | 7.75 | 4 | 6.25 | 3.226 |
| *Grb10^m/p^:Insr^-/-^* | 7.75 | 4 | 6.25 | 3.226 |
| TOTAL | 124 | 124 | 100 | 100 |

**Table S3.**

| *Grb10Δ2-4*: cross | Offspring stage | Litter size (range) | Litter size (mean) |
| --- | --- | --- | --- |
| *Igf1r* KO | PN1 | 1-13 | 4.8 |
| *Igf1r* KO | E17.5 | 5-15 | 9.8 |
| *Insr* KO | PN1 | 4-12 | 8.5 |
